# Development of a Novel Web Camera-Based Contact-Free Major Depressive Disorder Screening System Using Autonomic Nervous Responses Induced by a Mental Task and Its Clinical Application

**DOI:** 10.3389/fphys.2021.642986

**Published:** 2021-05-14

**Authors:** Batbayar Unursaikhan, Nobuaki Tanaka, Guanghao Sun, Sadao Watanabe, Masako Yoshii, Kazuki Funahashi, Fumihiro Sekimoto, Fumiaki Hayashibara, Yutaka Yoshizawa, Lodoiravsal Choimaa, Takemi Matsui

**Affiliations:** ^1^Graduate School of System Design, Tokyo Metropolitan University, Tokyo, Japan; ^2^Machine Intelligence Laboratory, School of Engineering and Applied Sciences, National University of Mongolia, Ulaanbaatar, Mongolia; ^3^BESLI Clinic, Tokyo, Japan; ^4^Graduate School of Informatics and Engineering, The University of Electro-Communications, Tokyo, Japan; ^5^Vital Lab, Ltd., Tokyo, Japan; ^6^RICOH Company, Ltd., Tokyo, Japan

**Keywords:** major depressive disorder, screening, heart rate variability, non-contact, remote photoplethysmography, autonomic nervous response, mental task

## Abstract

**Background:**

To increase the consultation rate of potential major depressive disorder (MDD) patients, we developed a contact-type fingertip photoplethysmography-based MDD screening system. With the outbreak of SARS-CoV-2, we developed an alternative to contact-type fingertip photoplethysmography: a novel web camera-based contact-free MDD screening system (WCF-MSS) for non-contact measurement of autonomic transient responses induced by a mental task.

**Methods:**

The WCF-MSS measures time-series interbeat intervals (IBI) by monitoring color tone changes in the facial region of interest induced by arterial pulsation using a web camera (1920 × 1080 pixels, 30 frames/s). Artifacts caused by body movements and head shakes are reduced. The WCF-MSS evaluates autonomic nervous activation from time-series IBI by calculating LF (0.04–0.15 Hz) components of heart rate variability (HRV) corresponding to sympathetic and parasympathetic nervous activity and HF (0.15–0.4 Hz) components equivalent to parasympathetic activities. The clinical test procedure comprises a pre-rest period (Pre-R; 140 s), mental task period (MT; 100 s), and post-rest period (Post-R; 120 s). The WCF-MSS uses logistic regression analysis to discriminate MDD patients from healthy volunteers via an optimal combination of four explanatory variables determined by a minimum redundancy maximum relevance algorithm: HF during MT (HF_*MT*_), the percentage change of LF from pre-rest to MT (%ΔLF_(Pre–R⇒*MT)*_), the percentage change of HF from pre-rest to MT (%ΔHF_(Pre–R⇒*MT)*_), and the percentage change of HF from MT to post-rest (%ΔHF_(MT⇒*Post–R)*_). To clinically test the WCF-MSS, 26 MDD patients (16 males and 10 females, 20–58 years) were recruited from BESLI Clinic in Tokyo, and 27 healthy volunteers (15 males and 12 females, 18–60 years) were recruited from Tokyo Metropolitan University and RICOH Company, Ltd. Electrocardiography was used to calculate HRV variables as references.

**Result:**

The WCF-MSS achieved 73% sensitivity and 85% specificity on 5-fold cross-validation. IBI correlated significantly with IBI from reference electrocardiography (*r* = 0.97, *p* < 0.0001). Logit scores and subjective self-rating depression scale scores correlated significantly (*r* = 0.43, *p* < 0.05).

**Conclusion:**

The WCF-MSS seems a promising contact-free MDD screening apparatus. This method enables web camera built-in smartphones to be used as MDD screening systems.

## Introduction

Major depressive disorder (MDD) has become one of the most serious mental health problems; globally, over 264 million people have MDD (James et al., 2018) and up to 15% of MDD patients show suicidal intent, particularly in young people (Kessler and Bromet, 2013; Whiteford et al., 2013). Indeed, the number of people who have experienced MDD during their lifetime was increased by nearly 20% for the last decade (Vos et al., 2016). Over 76% of people in developing countries and over 40% of people worldwide receive no relevant medical treatment for mental health disorders owing to lack of resources and field professionals (Wang et al., 2007). To encourage people to seek psychiatric support in the early stages of MDD, we previously developed a fingertip photoplethysmography (PPG)-based MDD screening system using stress-induced autonomic transient responses (Dagdanpurev et al., 2018).

SARS-CoV-2 infection can lead to serious symptoms such as respiratory disorders or multiple organ dysfunction (Dockery et al., 2020). SARS-CoV-2 is transmitted via oculus, nasus, and aditus facial mucosa (Yan et al., 2020), mainly through respiratory droplets and face-to-hand contact. Touching infected surfaces poses a potential risk, as people often instinctively touch their faces more than 20 times per hour (Kwok et al., 2015). Thus, we proposed a novel web camera-based contact-free MDD screening system (WCF-MSS).

Major depressive disorder is diagnosed by history taking and the criteria of the Diagnostic and Statistical Manual of Mental Disorders, Fifth Edition (DSM-5), supplemented by objective measures using biomarkers (Woods et al., 2014). Although rare, the possibility of incorrect diagnosis cannot be excluded if an examinee gives imprecise answers during history taking (Lépine et al., 1997). As an alternative to history taking, the WCF-MSS enables objective non-contact MDD screening using autonomic nervous system responses induced by a mental task (MT). Automatic nervous system activity can be evaluated using heart rate variability (HRV). The low frequency HRV component (LF: 0.04–0.15 Hz) corresponds to sympathetic and vagal tone activity and the high frequency HRV component (HF: 0.15–0.4 Hz) reflects parasympathetic activity. Previous research indicates that MDD patients have reduced HRV components at rest (Brunoni et al., 2013; Bassett, 2015; Carnevali et al., 2018). However, some researchers have reported a lack of correlation between HRV and MDD (Yeragani et al., 1991; Moser et al., 1998). These conflicting findings can be attributed to substantial individual differences in the autonomic activity of MDD patients during the rest state. Instead of using the rest state, we examined HRV-determined autonomic nervous responses induced by an MT (Sun et al., 2016). HRV is generally calculated using time-series interbeat intervals (IBI) measured by contact-based electrocardiography (ECG) or PPG. Previously, we developed HRV-based MDD screening systems using both ECG and fingertip PPG (Sun et al., 2016; Dagdanpurev et al., 2018). However, these conventional measuring techniques require contact-type sensors or electrodes. Long-term discomfort induced by contact-type devices affects the examinee’s autonomic nervous system activity (Westen, 2012; Culpepper et al., 2015).

To conduct non-contact MDD screening using a web camera, we used a minimum redundancy maximum relevance (MRMR) algorithm to determine the optimal combination of HRV-related autonomic nervous activity variables. To enable non-contact monitoring of HRV, we previously developed an HRV monitoring method using Doppler radar (Suzuki et al., 2008). The use of web camera-based contact-free remote PPG (rPPG) is another non-contact method of measuring HRV. Like PPG, rPPG uses optical methodology to sense heartbeat-induced arterial volume changes (Lewandowska et al., 2011). Unlike contact-based PPG, rPPG detects blood volume pulse (BVP) by tracking changes in facial luminance induced by microscopic arterial pulsations via a remote web camera (Poh et al., 2011; Wang et al., 2017). The WCF-MSS uses rPPG instead of Doppler radar. To optimize data processing procedures specialized for rPPG, we used techniques such as multiresolution analysis of the maximum overlap discrete wavelet transformation (MODWTMRA) to extract BVP from red-green-blue (RGB) color signals.

In the present study, we developed a novel WCF-MSS. Without the use of contact-type sensors or electrodes, the proposed system measures the examinee’s MT-induced autonomic nervous responses via an ordinary remote web camera. All web camera built-in devices, such as smartphones, tablets, and notebooks can potentially be used at home as MDD self-screening tools without the help of healthcare professionals. The WCF-MSS seems promising as a contact-free MDD screening tool that does not spread the COVID-19 infection.

## Materials and Methods

### Overview of the WCF-MSS

**FIGURE 1:**
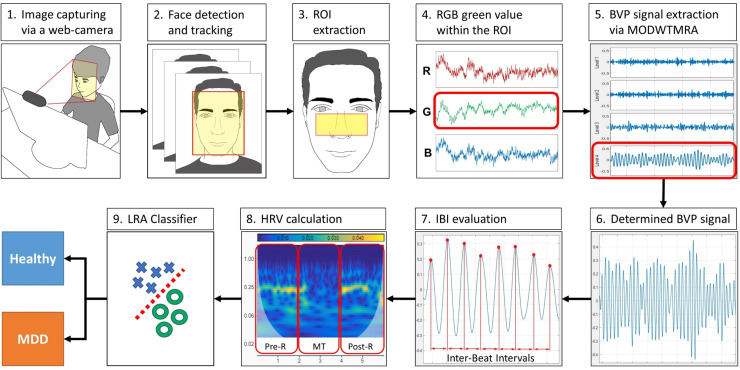
Block diagram of the WCF-MSS MDD screening procedure. (1) Web camera-based image capture before, during, and after the mental task (MT) at 30 frames/s. (2) Facial tracking for body movement cancelation. (3) Extraction of the region of interest (ROI). (4) RGB green signal within the ROI. (5) Extraction of pulse wave using wavelet transformation (MODWTMRA). (6) Blood volume pulse (BVP) signal determined by MODWTMRA. (7) Time-series interbeat intervals evaluation. (8) Heart rate variability (HRV) calculation for autonomic nervous activation monitoring during pre-rest, random number generation (RNG) MT, and post-rest. (9) Differentiation of MDD patients from healthy volunteers using logistic regression analysis (LRA). MDD, major depressive disorder; WCF-MSS, web camera-based contact-free MDD screening system.

The WCF-MSS MDD screening procedure is shown in Figure 1. The proposed system uses only a web camera for HRV measurement and a display for the MT paradigm. A web camera captures moving images of the subject’s face that reflect microscopic facial artery pulsations before, during, and after the MT. The system processes the captured facial images to extract heartbeat signals from facial luminance changes induced by arterial pulsations and processes the heartbeat signals to determine HRV-derived autonomic nervous activation induced by MT; this processing allows the system to differentiate MDD patients from healthy subjects. During the image-processing procedure, the system detects and tracks the subject’s face to adjust the size and location of the region of interest (ROI). The facial luminance of each frame (30 frames/s) within the ROI is determined by the green signal of the web camera’s RGB signals. During the signal-processing procedure, the BVP signal is determined from the green signal of the RGB signals via MODWTMRA. The LF (0.04–0.15 Hz) HRV component, the HF (0.15–0.4 Hz) HRV component, and the LF/HF are calculated using time-series IBI of the BVP signal for each measurement period; that is, the pre-rest (Pre-R), MT, and post-rest (Post-R) periods (Pre-R: 140 s, MT: 100 s, and Post-R: 120 s). Logistic regression analysis (LRA) was conducted to differentiate MDD patients from healthy volunteers via four explanatory variables related to autonomic activity.

### Image Processing

#### Facial Detection and Tracking

We used web camera-based rPPG methods to extract the BVP signal from the pixels of the human facial skin region. To perform trace detection of exact facial skin pixels from a captured image with body movements, we developed custom image-processing software using Python programming language (Python Software Foundation)^1^. The WCF-MSS detects the facial ROI associated with luminance alteration induced by arterial pulsations using the Haar cascade classifier from the Open Computer Vision (OpenCV) library (Bradski, 2000; Lienhart and Maydt, 2002; Viola and Jones, 2004). To track the ROI when the head is moving owing to posture-retaining balancing motions, the WCF-MSS uses the median flow object tracking algorithm (Kalal et al., 2010) from the OpenCV library. The median flow tracking algorithm keeps the ROI in the correct position during head movements.

#### Initializing and Adjusting the Region of Interest

**FIGURE 2:**
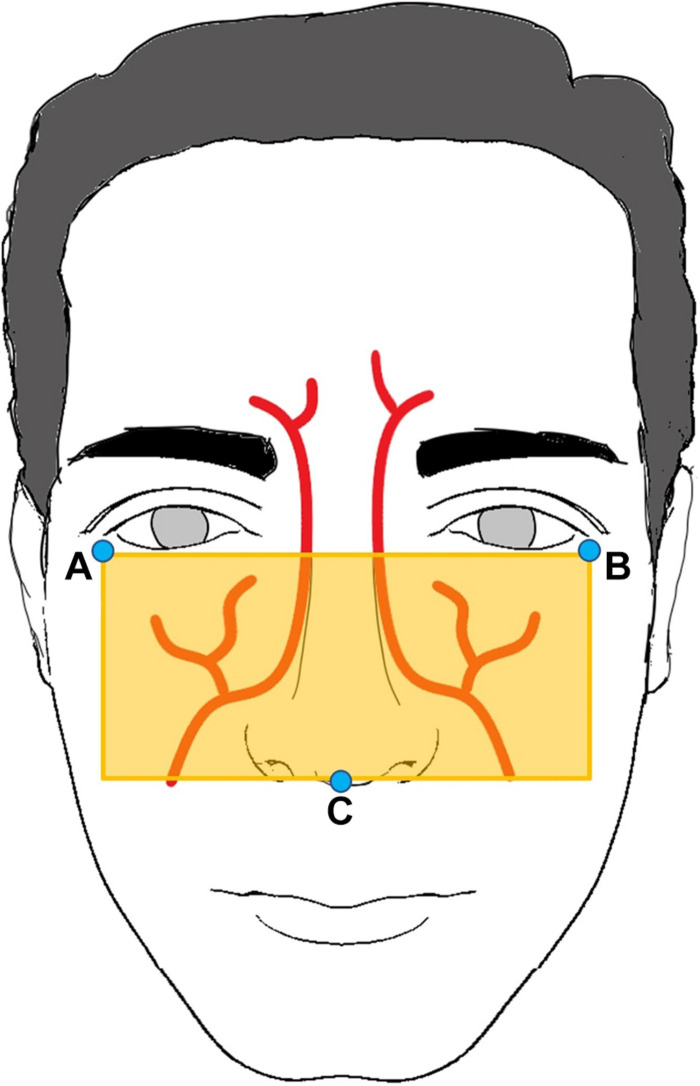
Region of interest determined by three landmark points: A, B, and C.

To extract the BVP signal efficiently, we determined the ROI using the anatomy of the facial arteries (von Arx et al., 2018). The ROI was determined using facial landmarks (A to C), as shown in Figure 2. Facial landmarks were determined using the neural network-based landmark-detection algorithm from the DLIB library (King, 2009). A 0.3 s window moving average filter was used to exclude artifacts and to determine the landmark points (A to C) unaffected by head motion.

### Signal Processing

#### Blood Volume Pulse Signal Extraction

The WCF-MSS extracts a BVP signal derived from averaged RGB green color signals within the ROI via MODWTMRA from MATLAB (The MathWorks, Inc., Natick, MA, United States). We used the MODWTMRA order 4 symlet wavelet filter with decomposition level 4 and band-pass filter (0.6–2.0 Hz), which extracts only cardiac-related signals.

#### Heart Rate Variability Analysis

To evaluate autonomic nervous activation induced by the MT, we used the HeartPy heart rate analysis toolkit (van Gent et al., 2018). The LF (0.04–0.15 Hz) component corresponds to HRV sympathetic and parasympathetic nervous activity and the HF (0.15–0.4 Hz) component reflects HRV parasympathetic activity. These were calculated from the time-series heartbeat intervals derived from BVP signals. LF and HF were calculated for the rest period before the MT (Pre-R), the period during the MT, and the rest period after the MT (Post-R). We determined 15 explanatory parameters for MDD classification: LF_(Pre–R)_, LF_(MT)_, LF_(Post–R)_, HF_(Pre–R)_, HF_(MT)_, HF_(Post–R)_, LF/HF_(Pre–R)_, LF/HF_(MT)_, and LF/HF_(Post–R)_, their deviations between Pre-R and MT, and their deviations between MT and Post-R.

### Major Depressive Disorder Screening Using Logistic Regression Analysis

To differentiate MDD patients from healthy volunteers, we used LRA from the MATLAB Machine Learning Toolbox. The linear equation determined by LRA is expressed as (*m* = 1 to 15):

(1) $$\log\frac{p}{1-p}=\beta_{0}+\beta_{1}\,x_{1}+\beta_{2}\,x_{2}+\ldots +\beta_{m}\,x_{m}$$

**FIGURE 3:**
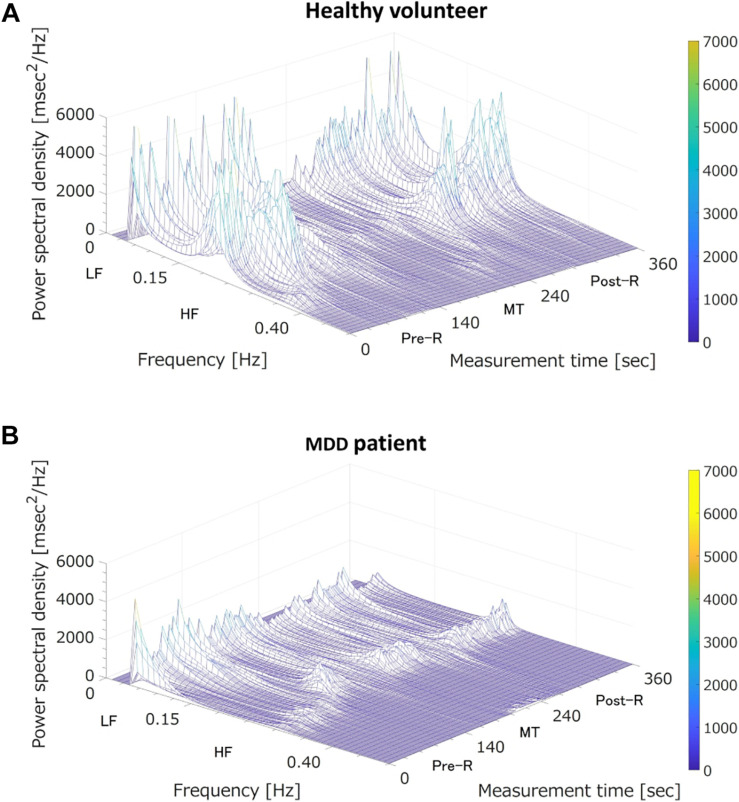
Three-dimensional mapping of power spectral densities of heart rate variability before the mental task (MT) (Pre-R), during the MT, and after the MT (Post-R). **(A)** A healthy volunteer; MT caused a large reduction in power spectrum density in the high frequency (HF) domain (corresponding to parasympathetic nervous activation). **(B)** A major depressive disorder (MDD) patient; MT induced no specific change in power spectrum density in the HF domain (corresponding to vagal tone activation). LF, low frequency.

Where $\log\frac{p}{1-p}$ is the predicted logit score, β_0_ is a constant, and β_1_…β_*m*_ are regression coefficients corresponding to the LRA explanatory variables of *x*_1…_*_*x*_m*. In our previous study, we used the three LRA explanatory variables LF, HF, and LF/HF, corresponding to three states (i.e., before MT, during MT, and after MT). In the present study, we identified 15 explanatory variables of the classifier (Sun et al., 2016; Dagdanpurev et al., 2018) suitable for non-contact measurement: LF_(Pre–R)_, LF_(MT)_, LF_(Post–R)_, HF_(Pre–R)_, HF_(MT)_, HF_(Post–R)_, LF/HF_(Pre–R)_, LF/HF_(MT)_, and LF/HF_(Post–R)_, their deviations between Pre-R and MT, and their deviations between MT and Post-R. Our previous study showed that MDD patients and healthy people showed different autonomic nervous responses induced by MT (Sun et al., 2016). As shown in Figure 3, the HF (corresponding to parasympathetic activity) of a healthy volunteer reduced substantially during MT, whereas the HF of an MDD patient showed no distinctive change during MT. To achieve an MDD screening accuracy equal to our previous study using ECG, we used the MRMR feature selection algorithm. This determined the optimal combination of LRA explanatory variables.

### Evaluation and Setting of the System

#### Participants

A total of 26 MDD patients and 27 healthy volunteers were recruited. The patients (aged 20–58 years; 16 males and 10 females) were from the BESLI clinic, Tokyo, and all had a diagnosis of MDD according to the DSM-5 criteria and were not on antidepressant medication. The control participants (18–60 years; 15 males and 12 females) were volunteers from Tokyo Metropolitan University and RICOH Company, Ltd., who had never received a psychological disorder diagnosis. All participants were instructed not to consume alcohol and coffee for 24 h before the study and not to smoke tobacco on the day of the study. Zung (1965) Self-Rating Depression Scale (SDS) scores were used to evaluate the severity of symptomology for all participants. An examinee with SDS cut-off score above 48 is suspected to having MDD (CIPS, 1996). SDS scores of all healthy volunteers in this study were below 48.

Regression analysis needs examinees ten times as many as the number of explanatory variables (Peduzzi et al., 1995). Our sample size of 53 examinees seems to be sufficient, while the number of explanatory variables is four. A chi-squared test revealed that there were no significant differences in the male/female ratio between MDD and healthy volunteer groups (*p* = 0.6). A chi-square test also revealed that there were no significant differences in age composition divided into three generations (a person younger than 25 years, a person aged 25 through 45 years, a person older than 45 years) in MDD and healthy volunteer groups (*p* = 0.2). A summary of the demographic of the healthy volunteers and the MDD patients is shown in Table 1.

**TABLE 1:** The demographic of the healthy volunteers and the MDD patients.

Classifications	Number	Sex and Age		Number	Average SDS scores
Healthy	27	Sex	Male	15	34.1
			Female	12	31.7
		Age	<25	10	28.9
			25–45	14	35.5
			45>	3	35.3
MDD	26	Sex	Male	16	52.7
			Female	10	52.6
		Age	<25	4	53.5
			25–45	18	51.7
			45>	4	56.0

This study was approved by the ethics committee of Tokyo Metropolitan University (approval number No 282) and BESLI clinic in Tokyo (approval number No 2018-001). All subjects provided written informed consent.

#### Study Protocol

**FIGURE 4:**
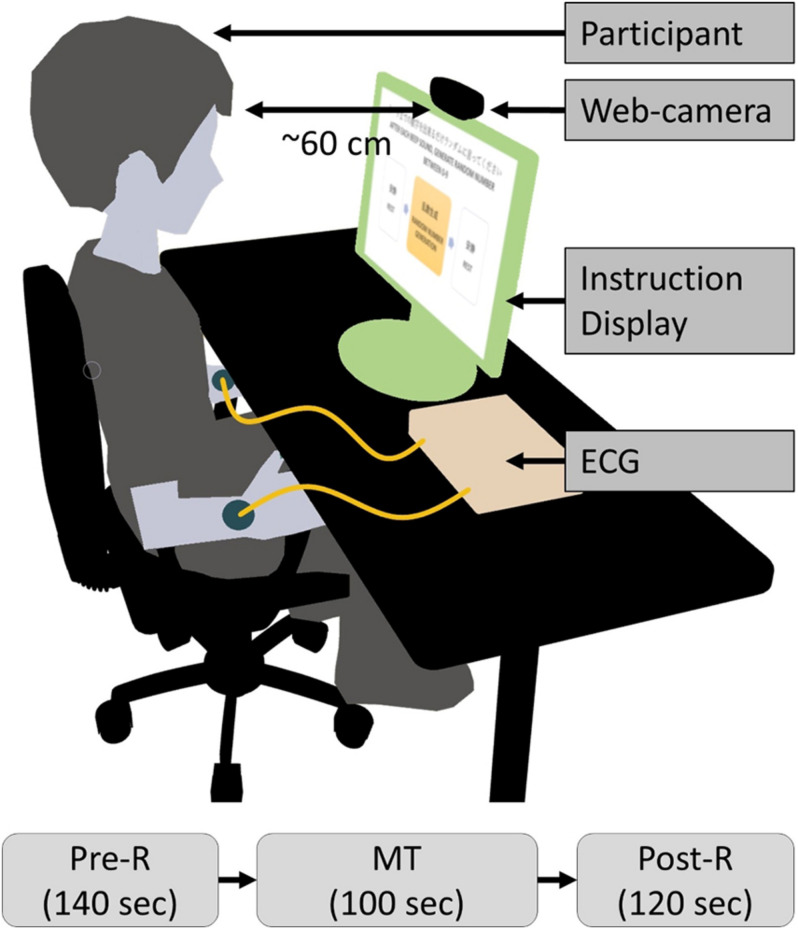
Diagram of measurement setup and study protocol. ECG, electrocardiogram; MT, mental task.

The clinical tests were conducted indoors, and primary lighting was the only source of illumination in the room. Participants were seated on a chair in front of a 22-inch display (providing visual instructions) at a distance of approximately 60 cm during the test, as shown in Figure 4. The study protocol of the clinical test procedure contained three periods: pre-rest (Pre-R; 140 s), random number generation MT (MT; 100 s), and post-rest (Post-R; 120 s), with a total duration of 360 s. Participants executed the tasks by following visual and audio instructions. During the Pre-R and Post-R periods, participants were instructed to relax. During the MT, participants generated random numbers between 0 and 9. The frequency of the random number generation was regulated by a displayed instruction and a “beep” once per s. The facial RGB signal was captured by a web camera (LogiCool HD Pro C920, Logitech International S.A., Lausanne, Switzerland) at a frame rate of 30 frames/s, a color pixel resolution of 1920 × 1080, and 256 tones. I-lead ECG readings were acquired simultaneously from both wrists at a sampling rate of 100 Hz as a reference.

### Statistical Analysis

Pearson’s correlation coefficient and Bland–Altman plots were used for statistical analysis of the correlation between the web camera measurement and the reference. The MRMR algorithm was used to determine the optimal combination of explanatory variables for MDD classification. The results from the LRA classification model were used to calculate the sensitivity, specificity, negative predictive value (NPV), and positive predictive value (PPV). A 5-fold cross-validation was performed to evaluate the accuracy of the LRA. The receiver operating characteristic curve was calculated to set the optimal cutoff point of the LRA model. Student’s *t*-test was conducted to statistically assess the logit scores of the LRA model.

## Results

**FIGURE 5:**
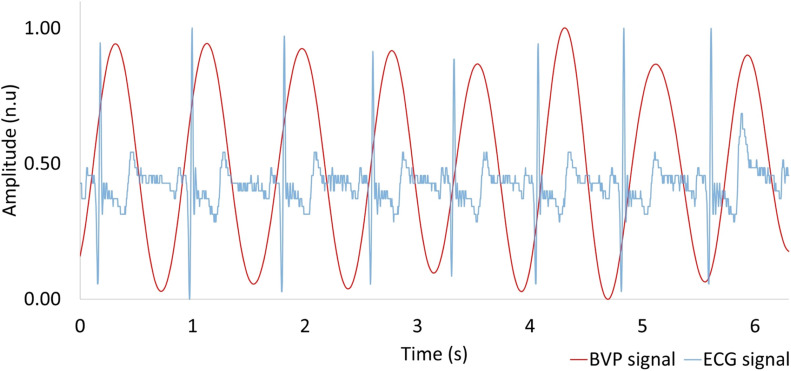
Comparison between heartbeat signal measured by a web camera (red) and reference ECG (blue). BVP, blood volume pulse; ECG, electrocardiogram.

Using the PPG technique described in section “Materials and Methods,” we extracted the BVP signal from the web camera RGB green signal via MODWTMRA. The BVP signal and the reference I-lead ECG are shown in Figure 5. The BVP signal was synchronous with the cardiac cycle as determined by the I-lead ECG. The pulse transmission time (time delay from ECG R-wave to BVP peak) was approximately 120 ms.

**FIGURE 6:**
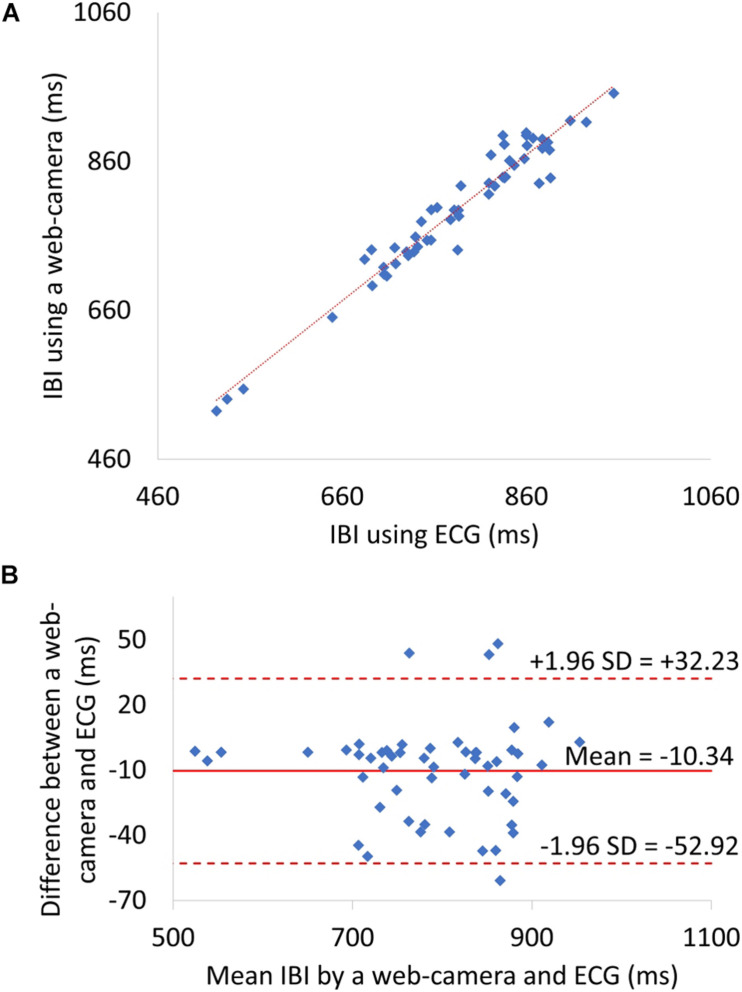
**(A)** A Scatter plots showing relationship between web camera-determined IBI and ECG-derived interbeat interval (IBI). **(B)** IBI Bland–Altman plot. ECG, electrocardiogram; SD, standard deviation.

The level of agreement between the web camera-based method and the reference ECG was assessed using the Pearson correlation coefficient (*n* = 53) and the Bland–Altman plot. Correlation scatter plots for IBI are shown in Figure 6A. The IBI determined by the web camera significantly correlated with that calculated from the reference ECG (*r* = 0.97, *p* < 0.0001). The root mean squared error of IBI was 24.05. Figure 6B shows the Bland–Altman plot for IBI determined by web camera and ECG. The 95% limits of agreement of IBI measurements ranged from −52.92 to −10.34 ms (standard deviation σ = 21.72).

**FIGURE 7:**
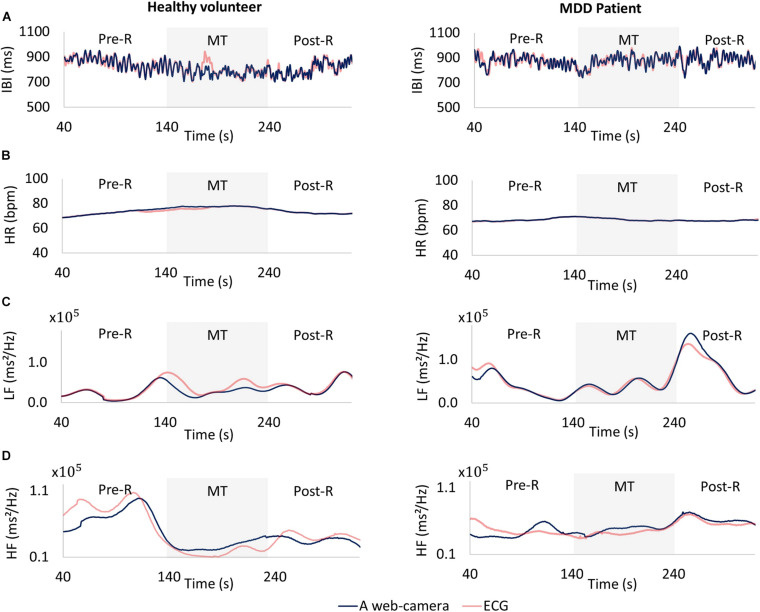
Comparisons between a web camera-derived parameter (blue) and those determined by reference ECG (yellow). **(A)** Interbeat intervals (IBI), **(B)** heart rate (HR), **(C)** low frequency (LF), **(D)** and high frequency (HF) during the measurement. Left: A healthy volunteer, Right: A major depressive disorder (MDD) patient. The HF of a healthy volunteer reduced substantially during a stressful MT (**D** left), whereas that of an MDD patient showed no distinctive changes (**D** right). ECG, electrocardiogram; MT, mental task.

The web camera-derived IBI of an MDD patient changed in the same manner as that determined by reference ECG (Figure 7A, right), whereas the web camera-derived IBI of a healthy volunteer showed small differences from that determined by reference ECG (Figure 7A, left). The web camera-derived heart rate of a healthy volunteer and an MDD patient changed in the same manner as those determined by reference ECG (Figure 7B). The web camera-derived LF of an MDD patient changed in the same way as that determined by reference ECG (Figure 7C, right), whereas the web camera-derived IBI of an MDD patient showed small differences from that determined by reference ECG (Figure 7C, right). The HF of a healthy volunteer reduced substantially during a stressful MT (Figure 7D, left), whereas that of an MDD patient did not show any distinctive changes (Figure 7D, right). The web camera-derived HF of an MDD patient and a healthy volunteer changed in a similar, but not identical, way to that of the ECG-determined LF and HF.

**FIGURE 8:**
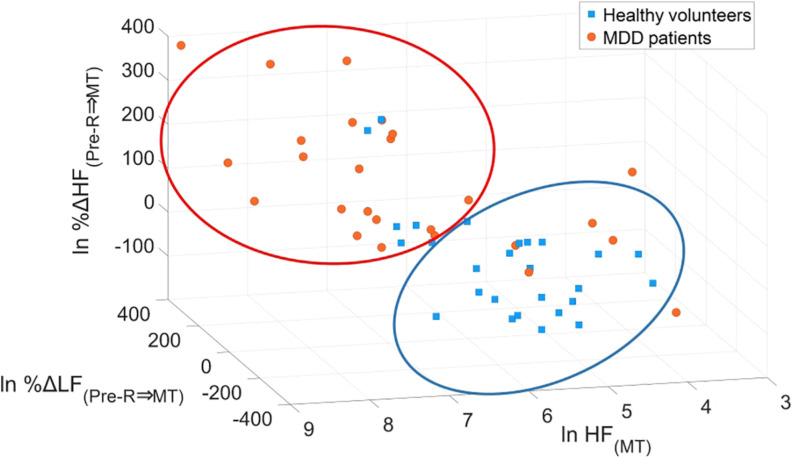
Scatter plots of logarithmic expression illustrating the three explanatory variables of HF_(MT)_, %ΔLF_(Pre–R⇒*MT)*_, and %ΔHF_(Pre–R⇒*MT)*_ in healthy volunteers and MDD patients. HF, high frequency; LF, low frequency; MDD, major depressive disorder; MT, mental task.

Minimum redundancy maximum relevance determined the following optimal combination of four variables: HF_(MT) (_*x*_1)_, %ΔLF_(Pre–R⇒*MT)* (_*x*_2)_, %ΔHF_(Pre–R⇒*MT)* (_*x*_3)_, and %ΔHF_(MT⇒*Post–R)*_ (*x*_4_) from the 15 potential LRA explanatory variables described above. Figure 8 shows the logarithmic expression of three explanatory variables: HF_(MT)_, %ΔLF_(Pre–R⇒*MT*)_, and %ΔHF_(Pre–R⇒*MT*)_ from the four explanatory variables determined by the MRMR algorithm for MDD patients and healthy volunteers. The combination of these three explanatory variables effectively screened for MDD patients.

**FIGURE 9:**
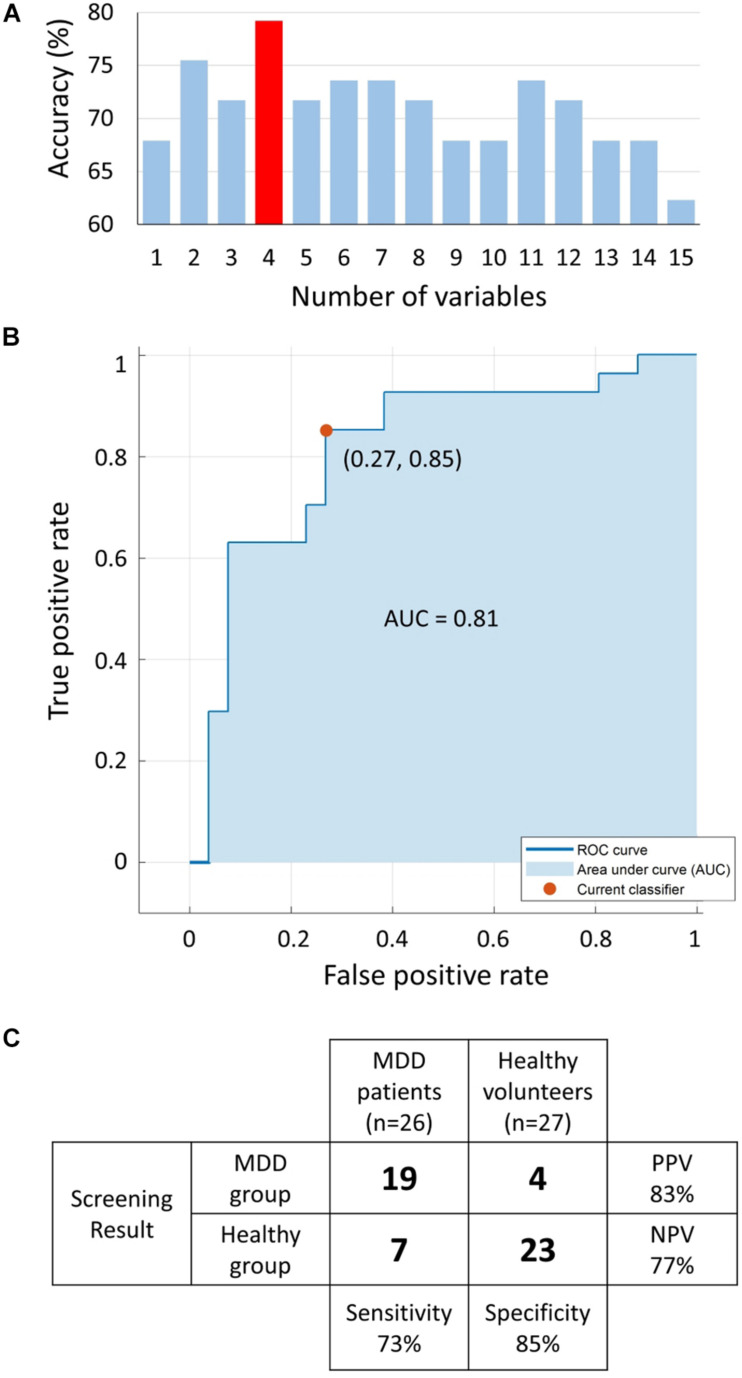
**(A)** Accuracy [mean of sensitivity, specificity, positive predictive value (PPV), and negative predictive value (NPV)] of logistic regression analysis (LRA) with 5-fold cross-validation as a function of number of explanatory variables; a combination of four explanatory variables exhibited the maximum accuracy. **(B)** The receiver operating characteristic (ROC) curve with area under the curve (AUC) of 0.81. **(C)** Confusion matrix of LRA corresponding to selected four explanatory variables to determine sensitivity, specificity, PPV, and NPV. MDD, major depressive disorder.

**FIGURE 10:**
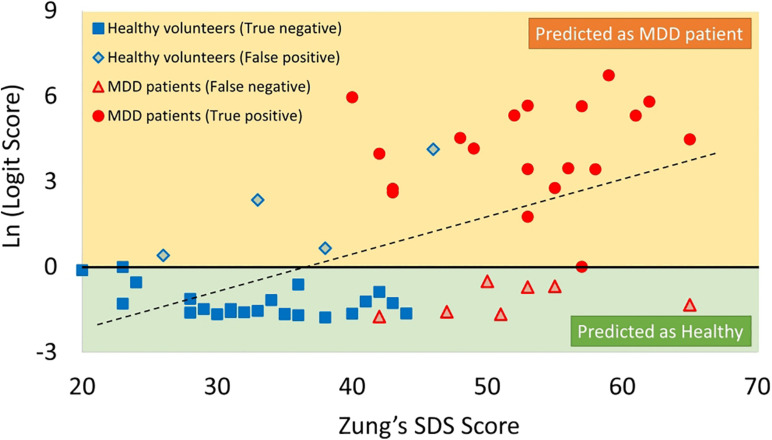
Scatter plots of correlation between SDS score and logit score in logarithmic expression. MDD, major depressive disorder; SDS, Self-Rating Depression Scale.

The MRMR algorithm determined the priority order of the previously mentioned 15 LRA explanatory variables. We used the combination of four high priority explanatory variables, as it showed the highest MDD screening accuracy (mean of sensitivity, specificity, PPV, and NPV, as shown in Figure 9A). The logit score (Figure 10; i.e., $\log\frac{p}{1-p}$) is expressed in the following equation using the optimal combination of four explanatory variables, where *p* is the probability and $\log\frac{p}{1-p}$ is the corresponding odds (Kessler and Bromet, 2013).

(2) $$\begin{array}{c} L\,o\,g\,i\,t\,S\,c\,o\,r\,e=l\,o\,g\,\frac{p}{1-p}\\ =-1.2895+0.0013\,H\,F_{M\,T}+0.0051\%\,\Delta \,L\,F_{\left(Pre-R\Rightarrow MT\right)}\\ -0.0001\%\,\Delta \,H\,F_{\left(Pre-R\Rightarrow MT\right)}-0.0004\%\,\Delta \,H\,F_{\left(MT\Rightarrow Post-R\right)}\\ L\,o\,g\,i\,t\,S\,c\,o\,r\,e≧0\Rightarrow S\,u\,s\,p\,e\,c\,t\,e\,d\,o\,f\,M\,D\,D\\ L\,o\,g\,i\,t\,S\,c\,o\,r\,e<0\Rightarrow H\,e\,a\,l\,t\,h\,y \end{array}$$

Receiver operating characteristic analysis was performed to determine the optimum cutoff point for the predicted logit scores of the LRA model to differentiate the two groups with an area under the curve of 0.81 (Figure 9B). The LRA confusion matrix with 5-fold cross-validation showed a sensitivity, specificity, NPV, and PPV of 73%, 85%, 77%, and 83%, respectively (Figure 9C).

The logit scores determined by equation (Kessler and Bromet, 2013) significantly correlated with SDS scores (*r* = 0.43, *p* < 0.05) (Figure 10).

## Discussion

To conduct MDD screening without risk of secondary exposure during the global COVID-19 pandemic, we developed a novel web camera-based non-contact MDD screening system (WCF-MSS) based on autonomic nervous activity response induced by MT. The non-contact WCF-MSS achieved 73% sensitivity and 85% specificity.

To achieve high screening accuracy, we measured not only HF_(MT)_, which reflects parasympathetic activation induced by MT, but also MT-induced percentage changes in HF and LF, which are related to sympathetic and parasympathetic nervous activities, using the variables %ΔHF_(Pre–R⇒*MT)*_, %ΔHF_(MT⇒*Post–R)*_, and %ΔLF_(Pre–R⇒*MT)*_.

The WCF-MSS could be used not only with MDD patients but also with non-MDD high-risk groups, as the WCF-MSS logit score significantly correlated with SDS scores. Early-stage WCF-MSS-based MDD screening may enable effective and low-cost treatment (Andrews et al., 2004; Rush et al., 2006; Trivedi et al., 2006; Cuijpers et al., 2009). The WCF-MSS could be used to exclude malingering, as it enables screening without history taking.

Owing to the COVID-19 pandemic, the use of telemedicine in clinical psychiatry has become increasingly important. The Internet-connected WCF-MSS could be used as a telemedicine terminal. Web camera built-in smartphones, tablets, and laptop computers could be used with the WCF-MSS as telemedicine devices. The use of the WCF-MSS in telemedicine enables automatic pre-examination before history taking by a psychiatrist.

One of the study limitations was the small dataset (53 subjects) compared with typical datasets in the medical classification field. To address this limitation, future work based on the present study should include larger datasets to improve the accuracy of the LRA classifier. One potential problem with the WCF-MSS is the difficulty of excluding the effect of sudden large movements. Further improvements are required to reduce such system artifacts.

In summary, the MT-induced autonomic nervous response-based contact-free WCF-MSS with 5-fold cross-validation achieved 73% sensitivity and 85% specificity. WCF-MSS designed for home use may be useful as a preliminary inspection tool for potential MDD patients who hesitate to go to psychiatry hospitals. The WCF-MSS appears a promising contact-free MDD screening tool that can be used without spreading COVID-19 infection.

## Data Availability Statement

The raw data supporting the conclusion of this article will be made available by the authors, without undue reservation.

## Ethics Statement

The studies involving human participants were reviewed and approved by The Ethics Committee of Tokyo Metropolitan University The Ethics Committee of BESLI clinic in Tokyo. The patients/participants provided their written informed consent to participate in this study. Written informed consent was obtained from the individual(s) for the publication of any potentially identifiable images or data included in this article.

## Author Contributions

BU and TM designed the research and wrote the manuscript. GS contributed to the writing of the manuscript. NT and FS supervised the medical aspects of MDD screening and performed clinical testing. GS, SW, MY, KF, FH, YY, and LC contributed to the image-processing and signal-processing methods. All authors reviewed the manuscript.

## Conflict of Interest

SW was employed by the company Vital Lab, Ltd., and MY and KF were employed by the company RICOH Company, Ltd. The remaining authors declare that the research was conducted in the absence of any commercial or financial relationships that could be construed as a potential conflict of interest.

## Footnotes

1 https://www.python.org

## Abbreviations

AUC: area under the curve
BVP: blood volume pulse
COVID-19: SARS-CoV-2
DSM-5: Diagnostic and Statistical Manual of Mental Disorders, Fifth Edition
ECG: electrocardiography
HF: high frequency
HRV: heart rate variability
IBI: interbeat intervals
LF: low frequency
LRA: logistic regression analysis
MDD: major depressive disorder
MODWTMRA: maximum overlap discrete wavelet transformation
MRMR: minimum redundancy maximum relevance
MT: mental task
NPV: negative predictive value
PPG: photoplethysmography
PPV: positive predictive value
RGB: red-green-blue
RNG: random number generation
ROC: receiver operating characteristic
ROI: region of interest
rPPG: remote photoplethysmography
SDS: Self-Rating Depression Scale
WCF-MSS: web camera-based contact-free MDD screening system.
